# Is ultra-hypo-fractionated radiotherapy more cost-effective relative to conventional fractionation in treatment of prostate cancer? A cost–utility analysis alongside a randomized HYPO-RT-PC trial

**DOI:** 10.1007/s10198-022-01467-5

**Published:** 2022-05-19

**Authors:** Sun Sun, Håkan Jonsson, Klas-Göran Salén, Mats Andén, Lars Beckman, Per Fransson

**Affiliations:** 1 Department of Epidemiology and Global Health, Umeå University, 90185 Umeå, Sweden; 2 Research Group Health Outcomes and Economic Evaluation, Department of Learning, Informatics, Management and Ethics, Karolinska Institutet, Stockholm, Sweden; 3 Center for Cancer Control and Policy Research, Shandong University, Jinan, Shandong Province China; 4 Department of Oncology, Kalmar Hospital, Kalmar, Sweden; 5 Department of Oncology, Sundsvall Hospital, Sundsvall, Sweden; 6 Department of Nursing, Umeå University, Umeå, Sweden; 7 Department of Radiation Sciences, Umeå University, Umeå, Sweden

**Keywords:** Cost–utility analysis, Prostate cancer, Within-trial economic evaluation, Radiotherapy, Intermediate-to-high-risk cancer, I19

## Abstract

**Background:**

Economic evidence for comparing low fraction with ultra-hypo fractionated (UHF) radiation therapy in the treatment of intermediate-to-high-risk prostate cancer (PC) is lacking, especially in Europe. This study presents an economic evaluation performed alongside an ongoing clinical trial.

**Aim:**

To investigate up to 6 years’ follow-up whether conventional fractionation (CF, 78.0 Gy in 39 fractions, 5 days per week for 8 weeks) is more cost-effective than UHF (42.7 Gy in 7 fractions, 3 days per week for 2.5 weeks inclusive of 2 weekends) radiotherapy in treatment for patients with intermediate-to-high-risk PC.

**Method:**

HYPO-RT-PC trial is an open-label, randomized, multicenter (10 in Sweden; 2 in Denmark) phase-3 trial. Patients from Sweden (CF 434; UHF 445) were included in this study. The trial database was linked to the National Patient Registry (NPR). Costs for inpatient/non-primary outpatient care for each episode were retrieved. For calculating Quality-adjusted life years (QALYs), the EORTC QLQ-C30 questionnaire was mapped to the EQ-5D-3L index. Multivariable regression analyses were used to compare the difference in costs and QALYs, adjusting for age and baseline costs, and health status. The confidence interval for the difference in costs, QALYs and incremental cost-effectiveness ratio effectiveness ratio (ICER) was estimated by the bootstrap percentile method.

**Results:**

No significant differences were found in ICER between the two arms after 6 years of follow-up.

**Conclusion:**

The current study did not support that the ultra-hypo-fractionated treatment was more cost-effective than the conventional fraction treatment up to the sixth year of the trial.

**Supplementary Information:**

The online version contains supplementary material available at 10.1007/s10198-022-01467-5.

## Background

Radiotherapy is one of the mainstays of treatment for intermediate and high-risk prostate cancer (PC) with conventional fractionation (CF) schedule of 1.8–2.0 Gy fractions. In recent years, studies have suggested that PC has a high fractionation sensitivity and, therefore, moderate hypofractionation (MHF) qualifies as a viable option with similar outcome and toxicity as CF schedules [1–4].

Recently data from the HYPO-RT-PC randomized trial showed that ultra-hypo-fractionation (UHF) with a fraction size of 6.1 Gy per fraction delivered in seven fractions in 2.5 weeks is safe and comparable to conventional radiotherapy [5, 6]. UHF, therefore, might be a candidate to reduce the overall treatment time and also increase patient convenience, thus might be an attractive treatment alternative. Apart from clinical effectiveness in health outcomes including quality of life (QoL), it is also relevant to consider costs and effectiveness jointly, to improve treatment guidelines and inform decision-makers.

Economic evaluations have been performed to compare radiation therapies for PC [7–9], however, only a few studies have compared low/conventional fraction treatment with ultra-hypo-fraction treatment [10–12], and just one study was from Europe [12]. All studies applied the Markov model*;* however, key model parameters such as progression-free survivals and toxicity rates were all taken from non-randomized trials with short follow-ups. This might be because UHF is a rather new intervention, limited information from RCTs were available at that time. Furthermore, the Markov models assume equal efficacy, utility, costs and transition probability for patients in the same health state, which brings challenges to including individual patient characteristics into the analyses. Therefore, a health economic evaluation alongside an RCT could provide timely evidence, as well as information about how patient characteristics are associated with costs and health outcomes.

The current study is based on the HYPO-RT-PC trial, details regarding the trial have been published elsewhere [5, 6]. In brief, the HYPO-RT-PC trial is a Scandinavian randomized phase 3 trial where men with PC were randomly assigned to either CF or UHF. As the UHF requires fewer clinical visits for radiotherapy relative to CF, we expected to see lower costs in the UHF arm. As existing clinical evidence suggested that there was no difference in clinical outcomes between the two arms [5, 6], we expected no differences in QALYs between the arms. To the best of our knowledge, this study is the first one to present the cost–utility analysis (CUA) alongside an ongoing randomized clinical trial that compares UHF with CF radiotherapy.

## Aim

To investigate alongside a clinical trial in Sweden, whether ultra-hypo-fractionated radiotherapy is more cost-effective than conventional fractionation in the treatment of patients with intermediate-to-high-risk prostate cancer up to 6 years’ follow-up time.

## Materials and methods

### Design and participants in the trial

This study was designed as a cost–utility analysis from a health care perspective, based on the HYPO-RT-PC study [5]. The HYPO-RT-PC study is an open-label, randomized, multicenter, non-inferiority phase 3 trial performed in 10 centres in Sweden and 2 in Denmark. Briefly, male patients aged below 75 years, with a histologically verified intermediate-to-high-risk PC, were eligible for radiotherapy within the study. No endocrine treatment was permitted. The patients had to be lymph node-negative and with no evidence of metastases. Patients were randomly assigned in a 1:1 ratio to either CF or UHF. Patients were recruited consecutively at trial centres between July 1, 2005, and Nov 4, 2015, and were followed-up with questionnaires up to 15 years or (3, 6, 12 months, 2, 4, 6 and 10 years) until metastatic progression or death.

For the present study, only patients from Sweden at the study time (up to six years’ follow-up) were included, all completed self-assessed questionnaires at the trial entry (baseline). Baseline and RT-end questionnaires were administered on-site by the clinic while the remaining questionnaires were posted to patients by regular mails. A reminder letter was sent to those who did not respond within 30 days. All patients provided written and verbal informed consent. The study was approved in Sweden by the Ethics Committee in Umeå (reference number 03-513, 2003-12-23) and in Denmark by the Central Denmark Region Committees on Health Research Ethics (M-20090180, 2009-11-19). The study protocol is available online (https://www.umu.se/en/research/groups/hypo-rt-pc). It is registered at the ISRCT register (Trial no: ISRCTN45905321, https://doi.org/10.1186/ISRCTN45905321).

### Treatments

Patients in the CF arm received 78.0 Gy in 39 fractions (5 days per week for 8 weeks) whereas patients in the UHF arm received 42.7 Gy in seven fractions (3 days per week for 2.5 weeks inclusive of 2 weekends), prescribed as the mean PTV dose.

### Health outcomes

QoL was measured by the European Organization for Research and Treatment of Cancer Quality-of-Life Questionnaire–Core 30 (EORTC QLQ-C30) [13]. EORTC-C30 is a cancer-specific instrument, which includes five functional scales (cognitive; emotional; physical; role; and social functioning), three symptom scales (fatigue; nausea/vomiting; and pain), a global health status scale, and five single items assessing additional symptoms (appetite loss; constipation; diarrhoea; dyspnea; and sleep disturbance) and perceived financial impact) [14]. Details of the outcomes based on EORTC-QLQ-C30 have been reported elsewhere [6].

For calculating Quality-adjusted life years (QALYs), and health utility, the EORTC-QLQ-C30 was mapped to EQ-5D-3L, based on the existing mapping algorithm [15]. QALYs were calculated using the area under the curve (AUC) method: EQ-5D index at each time point was represented as data points, which were first joined by straight lines to define the “curve”, then AUC was calculated by adding the areas under the curve between each pair of consecutive observations [16]. Imputation for missing data on QoL was performed in R using the “mice” package [17]. A two-step multiple-imputation method was applied [18]: first, in case of missing item on the EORTC-QLQ-C30 items (ordered categorical variables), the proportional odds imputation method was used to impute the level of answering on each EORTC-QLQ-C30 item, using age, sex, treatment group, follow-up time and answering on the non-missing EORTC-QLQ-C30 items, the imputed EORTC-QLQ-C30 were then used for calculating EQ-5D index; in case of the missing form i.e., due to patients lost to follow-up (without progression or dead), the predictive mean matching imputation method was used to predict the EQ-5D index, using age, treatment group, follow-up time and non-missing EQ-5D index as predictors; for patients who died during the trial, their EQ-5D index was assigned as 0; for patients progressed, the EQ-5D index was considered as a 17% drop from the latest report, which was indicated by a previous study in Sweden [19].

### Costs

The HYPO-RT-PC trial data was linked via the unique personal identification number with the Swedish National Patient Registry (NPR). Costs for inpatient/non-primary outpatient care for each episode at the individual level were retrieved from NPR, and the diagnostic-related group (DRG) costs were used. Methods for estimating costs have been applied in a previous study in Sweden [20]. The NPR database is a national patient administrative dataset (contains information on outpatient, inpatient, and psychiatric care), managed by the Swedish National Board of Health and Welfare (SNBHW, Socialstyrelsen in Swedish). The Swedish version of the International Statistical Classification of Diseases and Related Health Problems Version 10 (ICD-10) called ICD-10-SE is used for coding primary and secondary diagnoses, which is mandatory for reporting [21].

Costs were reported in Swedish kronor (SEK) (1€ = 10.83 SEK, exchange rate on 2021-03-04). The costs were categorised based on the primary diagnosis (Swedish ICD-10) and further grouped according to the ICD-10 grouping (Supplementary material S1). For the current study, two types of summary costs were calculated: costs directly related to PC and its treatment (ICD10: C639, N00-99, Z00-99; cost 1); all costs regardless of which primary diagnosis was associated (cost 2). As the 6th year follow-up was conducted approximately 6 years from the randomizing date, for costs calculation, costs that occurred within 6.5 years were considered relevant.

As not all costs occurring during the trial were directly related to PC, adjusting for costs that occurred before baseline was appropriate. The mean cost occurring within 2 years before baseline (cost3) was estimated (2855 SEK, CF; 3056 SEK, UHF; *p* value 0.643). The distribution of cost3 was highly skewed and with a large number equal to “0”, i.e., no cost. To facilitate the analyses, the mean of cost3 for the non-zero observation was accessed (11,962 SEK), and this number together with “0” was applied as the cutting-off points for dividing cost3 into three groups (Supplementary material S2). As the majority of the participants were retired, costs due to productivity loss were not considered.

### Cost–utility analysis

Cost effectiveness was accessed based on actual patient-level data, evaluated by relating differences in total costs to differences in QALYs between the two treatment arms. The incremental cost effectiveness ratio (ICER) is the ratio between the difference in mean total costs (ΔC) and the difference in health effect (ΔE) of two groups: ICER = ΔC/ΔE. The confidence interval (CI) for ICER was estimated by the bootstrap percentile method, generating 1000 replications of each ratio. These ΔC/ΔE ratios were depicted in a cost-effectiveness plane to evaluate the simultaneous dispersion of cost and health effects and to infer the likelihood of UHF being cost effective.

### Statistical analysis

The distribution of cost data is usually skewed. The distribution was plotted; mean and standard deviation (SD) and quartiles were calculated, for the CHF and UHF arm, respectively (S1). For comparison of the means of costs and QALYs, the two-sample *t* test, and bootstrap were used. In cost analysis, the Generalized linear models (GLM) assuming gamma distribution with log and identity links were commonly applied [22, 23]. Therefore, in the initial step, the same assumption was adopted. In the next step, a Modified Park test [24] was performed to select the appropriate distribution family. We used the identity link function for easy interpretation. For QALYs analysis, linear regression with the Ordinary Least Square (OLS) method was applied. For all analysis, three types of models were tested: in model 1, only the treatment group were applied as the independent variable; in Model 2, age was adjusted; in Model 3, both age and cost occurring 2 years before baseline were adjusted. All analyses were conducted using R.4.0.2 [25].

## Results

The number of patients by follow-up for both treatment arms is presented in (Table 1). There were 434 patients in the CF arm and 445 in the UHF arm. No differences in mean age between the two arms were found: CF (mean 69, SD 4.9) and UHF (mean 69, SD 5.2). The follow-up rate across time was similar between the two arms as well, 80% of the patients were followed at the end of treatment, and decreased to about 30% at the end of the sixth year of the trial (30% in CF, 27% in UHF).

**Table 1:** Number of patients by follow up time, by treatment arm

	CF		UHF	
	*n*	%	*n*	%
Baseline	434	100	445	100
End of treatment	373	86	372	84
3 month	262	60	266	60
6 month	275	63	275	62
1 year	274	63	268	60
2 year	299	69	318	71
4 year	231	53	239	54
6 year	131	30	121	27

The mean of EQ-5D index at each follow-up, as well as QALYs (from baseline up to the sixth year) for both treatment arms, are presented in (Table 2). EQ-5D index was significantly higher in CF (0.617) relative to UHF at the baseline (UHF 0.605; *p* = 0.028), but no significant differences in EQ-5D index at the other follow-ups, or QALYs were found.

**Table 2:** Mean EQ-5D index at each follow-up, and mean QALYs (baseline to 6th year), and *p* value for comparing the difference (Student’s *t* test), by treatment arm

	EQ-5D index		
	CF	UHF	*p* value
Baseline	**0.617**	**0.605**	**0.028**
End of treatment	0.598	0.590	0.189
3 month	0.607	0.604	0.608
6 month	0.607	0.604	0.518
1 year	0.600	0.600	0.900
2 year	0.594	0.586	0.298
4 year	0.581	0.568	0.123
6 year	0.577	0.567	0.251
Mean QALYs (baseline to the 6th year)	3.535	3.482	0.193

Statistically significant values are in bold (*p* < 0.05)

Mean costs for each follow-up and the entire period (baseline to 6th year) by detailed ICD category and treatment arm are reported in (Table 3). For those costs only related to PC (cost 1), only for period 8 weeks–3 months, and 3–6 months, UHF had significantly higher costs relative to CF. For costs including all diseases, UHF had significantly higher costs relative to CF at 1–2 years period, 6–6.5 years period, and for the entire period (baseline to 6 years).

**Table 3:** Mean costs (SEK) for each follow-up and the entire period (baseline to 6th year), and *p*-value for comparison of the difference (*t*-test), by treatment arm and follow-up time

	Cost1 (including costs for C619, N00-99 and Z00-99)			Cost2 (including costs for all the ICD categories)		
	CF	UHF	*p* value	CF	UHF	*p* value
0–2.5 weeks	13,263	12,108	0.307	14,623	12,316	0.067
2.5–8 weeks	7815	8598	0.297	9559	11,309	0.455
8 weeks-3 month	**6555**	**8568**	**0.020**	9687	18,364	0.084
3–6 month	**8868**	**11,432**	**0.019**	14,083	18,379	0.375
6 month-1 year	11,177	12,050	0.377	22,104	19,247	0.582
1–2 years	12,411	13,721	0.260	**24,228**	**30,361**	**0.007**
2–4 years	14,798	14,202	0.672	33,512	34,499	0.149
4–6 years	16,430	18,234	0.409	32,977	49,021	0.073
6–6.5 years	15,756	22,117	0.167	**30,548**	**58,689**	**0.011**
Baseline − 6.5 years	106,126	126,471	0.139	**328,082**	**418,642**	**0.021**

Statistically significant values are in bold (*p* < 0.05)

Mean costs by ICD category and treatment arm are reported in Supplementary material S1. UHF had significantly higher costs relative to CF in diseases of the circulatory system (I00-99, the difference was 8029 SEK, *p* = 0.037) and the “Z00-99” difference was 1772 SEK, *p* = 0.043). For costs related to PC (C619, N00-99 and Z00-99), the differences were very small. This analysis was further stratified by follow-up time (Supplementary Fig. 1), the curve for C619 ( PC) and Z00-00 were almost overlapping between the two arms, for N00-99 (diseases of the urinary, and genital organs), UHF had higher costs relative to CF, but the differences were small compared with costs in some of the other ICD categories such as L00-99, A00-B99. The large difference in costs between the two arms was mainly contributed by costs not directly related to PC.

The distribution of cost was reported in Supplementary Fig. 2, as expected, the plotting of the cost data was typically right-skewed. Multivariable analysis on costs is reported in (Table 4). Results from the modified Park test was more favour to Gaussian distribution, although both Gamma and Gaussian distribution gave similar results. For costs directly related to PC (cost1), no significant differences were found between the two arms across all models. For costs related to all diseases (cost2), coefficients for UHF were higher relative to CF across all models except Model 3 under Gamma distribution.

**Table 4:** Multivariable analysis (Generalized linear model) on costs (SEK)

	Cost 1, including costs for prostate cancer and related disease (C619, N00-99 and Z00-99)											
	Gamma distribution						Gaussian distribution					
	Model 1		Model 2		Model 3		Model 1		Model 2		Model 3	
	Coefficients	*p* Value	Coefficients	*p* Value	Coefficients	*p* Value	Coefficients	*p* Value	Coefficients	*p* Value	Coefficients	*p* Value
Intercept	**106,127**	**< 0.001**	**105,513**	**< 0.001**	**110,027**	**< 0.001**	**106,127**	**< 0.001**	**106,234**	**< 0.001**	**108,838**	**< 0.001**
UHF^a^	20,345	0.135	21,403	0.097	15,922	0.203	20,345	0.140	19,902	0.149	19,457	0.159
Age^b^	**–**	–	− 1780	0.182	− 1222	0.331	−	–	− 1718	0.208	− 1642	0.230
Cost3 (costs accured 2 years prior to baseline^c^												
1–11,962	–	–	–	–	4155	0.812	–	–	–	–	5118	0.773
11,963–63,030	–	–	–	–	**− 49,436**	**0.004**	–	–	–	–	− 57,535	0.053
AIC	22,230		22,225		22,213		23,966		23,966		23,966	

	Cost 2, including costs for all the ICD categories											
	Gamma distribution						Gaussian distribution					
	Model 1		Model 2		Model 3		Model 4		Model 5		Model 6	
	Coefficients	*p* Value	Coefficients	*p* Value	Coefficients	*p* Value	Coefficients	*p* Value	Coefficients	*p* Value	Coefficients	*p* Value
Intercept	**328,083**	**< 0.001**	**330,640**	**< 0.001**	**331,647**	**< 0.001**	**328,083**	**< 0.001**	**327,791**	**< 0.001**	**327,440**	**< 0.001**
UHF^a^	**90,560**	**0.020**	**85,553**	**0.029**	73,148	0.058	**90,560**	**0.021**	**91,772**	**0.020**	**88,785**	**0.024**
Age^b^	–	–	4166	0.252	4729	0.169	–	–	4694	0.228	5075	0.193
Cost3 (costs accured 2 years prior to baseline^c^												
1–11,962	–	–	–	–	57,791	0.299	–	–	–	–	51,576	0.309
11,963–63,030	–	–	–	–	− 104,700	0.074	–	–	–	–	− 135,386	0.109
AIC	24,270		24,270		24,266		25,806		25,806		25,806	

Statistically significant values are in bold (*p* < 0.05)

Reference group

^a^CHF

^b^Centred at mean age 69

^c^No cost (0) reference group

Multivariable analysis on QALYs is reported in (Table 5). No significant differences were found between the two arms across all models.

**Table 5:** Multivariable analysis (linear regression) on QALYs

	QALYs					
	Model 1		Model 2		Model 3	
	Coefficients	*p* Value	Coefficients	*p* Value	Coefficients	*p* Value
Intercept	**3.536**	**< 0.001**	**3.536**	**< 0.001**	**3.947**	**< 0.001**
UHF^a^	− 0.053	0.186	– 0.055	0.175	− 0.010	0.773
Age^b^	–	–	− 0.005	0.173	− 0.006	0.106
Baseline EQ-5D index	–	–	–	–	**1.103**	**< 0.001**
Adjusted Rsq	0.001		0.002		0.254	

Statistically significant values are in bold (*p* < 0.05)

Reference group

^a^CHF

^b^Centred at mean age 69

Results from the bootstrap of ICER were in line with the findings from the multivariable analysis (Table 6, Supplementary Fig. 3), that no significant difference was found in cost1 (95% CI: − 4211, 48,933) and QALYs (95% CI: − 0.1315, 0.0273) between the two treatment arms; UHF had significantly higher costs relative to CF in cost2 (95% CI: 10,286, 170,811 SEK). The 95% CI for ICER were (− 3,913,487, 2,309,086) SEK/QALY for cost1,

**Table 6:** Confidence intervals (CI) for cost (SEK), QALYs and incremental cost-effectiveness ratio (ICER) from bootstrap

	Cost 1 including costs for prostate cancer and related disease (C619, N00-99 and Z00-99)		Cost 2including costs for all ICD categories	
	CI-low	CI-up	CI-low	CI-up
Δ Costs	− 4211	48,933	10,286	170,811
Δ QALY	− 0.1315	0.0273		
ICER (ΔCost/ ΔQALY)	− 3,913,487	2,309,086	− 15,818,470	11,743,449

and (− 15,818,470, 11,743,449) SEK/QALY for cost2, both across “0” suggesting non-significant differences in cost effectiveness between the two arms.

## Discussion

The current study did not support that UHF was more cost-effective than CF in the treatment of intermediate-to-high-risk PC in Sweden. Clinical studies have shown that UHF would be comparable to CF from a biochemical control standpoint [1, 2, 4, 5, 26]. However, at the same time, the side effects might be more pronounced in the high fraction treatment, which could potentially negatively affect the QoL of patients and cause an increase in costs. The present study also showed lower QoL of UHF relative to CF at post-treatment follow-ups, however, the results were not significant. A possible explanation could be the sample size for the RCT was estimated based on detecting differences in clinical outcomes, which might not be sufficient for detecting differences in QoL. To the best of our knowledge, there was no economic evaluation comparing the treatment options which were the same as the current study, as the clinical trial for the current study is among the first ones of its kind [5]. Thus, direct comparison with similar studies was not feasible at this stage. We could only relate our study to economic evaluations based on other hypo-fractionated treatment schedules and techniques. The study by Hodges et al. compared stereotactic body radiation therapy (SBRT) with intensity-modulated radiation therapy (IMRT), suggested that SBRT might have a potential value, however, the cost-effectiveness was highly sensitive to QoL outcomes, that a decrease in QoL of 4% or a decrease in efficacy of 6% would lead to the conclusion that the SBRT has not favoured anymore [10]. The study by Sher et al. advocate the cost-effectiveness of SBRT over IMRT, but the authors acknowledged that the analysis was based entirely on non-randomized trials, and the parameters for the SBRT were immature as recurrence and toxicity risks were at relatively short follow-up (3 years) [11]. Both studies were conducted in the US, and costs were estimated from the payer’s perspective (Medicare). There are substantial differences in health systems between US and European countries, as the latter are mainly publicly funded with almost universal coverage, while the former were insurance-based. Therefore, the results based on cost-effectiveness analysis (CEA) studies in the US cannot be generalized in Europe. The study by Zemplényi et al. was conducted in Hungary, which compared high-dose IMRT and hypo-fractionated IMRT versus conventional dose three-dimensional radiation therapy (3DCRT). The author concluded that compared to 3DCRT, both IMRT and HF-IMRT resulted in more health gains at a lower cost in a mixed hypothetical cohort consisting of low-, intermediate- and high-risk patients. However, the cost-effectiveness was less for low risk than for intermediate- or high-risk patients. To summarize, so far the above three CEA studies were all based on Markov models, which assume equal efficacy, utility, costs and transition probability for patients in the same health state. Furthermore, key model parameters such as progression-free survivals and toxicity rates were all from non-randomized trials with short follow-ups. Our study is based on an ongoing multicenter randomized trial, with up to 6 years of follow-up, and individual varieties in costs and health outcomes were considered in the analyses, which provide a complementary picture for this important issue. A follow-up study will be carried out based on longer follow-up (> 6 years) data in the future.

QALY’s were somewhat lower in the UHF arm relative to the CF arm during the post-treatment period but no significant differences in QALYs were found between the two arms after 6 years. These findings were in line with earlier published clinical findings from the HYPO-RT-PC trial [5, 6]. There were no significant differences in overall survival and cumulative incidence of PC death between the treatment arms [5]. Furthermore, results showed no difference at the 6 year follow-up in the incidence of clinically relevant deterioration between the groups for overall QoL and cancer-related symptoms [6]. Although the UHF arm required less frequent radiation relative to the CF arm, the difference in PC treatment-related costs was not different between the two arms. It might reflect the fact that early side-effects were more pronounced in UHF than in CF. For example, there was an increase in urinary toxicity at 1 year follow-up as well as more bowel toxicity in the UHF compared with the CF [5]. The proportion of patients with clinically relevant deteriorations at the end of radiotherapy was also significantly higher in the UHF arm than in the CF arm, such as stool frequency, rush to the toilet, flatulence, bowel cramp, mucus, blood in stool, and limitation in daily activity[6].

Sweden is among the few countries where national databases for costs are available for all inpatient/outpatient episodes for all residents. However, at the same time, such rich information also brings challenges in analyses. For example, it was hard to decide which costs to be included. We used two different cost summary measures: one for prostate-related (cost1) and one including all diseases (cost2). This might give different aspects when looking at costs in the real world. The categorization of costs was based on the clinical expert’s (co-author: PF) suggestion. Following the societal perspective, it might be more appropriate to address all the costs regardless of their relevance with the disease/treatment. If one uses cost2 only, one might underestimate the impact of the disease on society. However, from a clinician point of view, the disease-related costs are more relevant. Also when the study involves medical services that are unrelated to the disease, it may be difficult to detect the influence of the treatment on total health-care [27]. In this study, we present both results, which is in line with the ISPOR guidelines [27]. To the best of our knowledge, this is the first attempt to demonstrate the cost components due to different disease categories for PC patients.

Diagnose-related group (DRG) for cost calculation can be seen as a quite good measure [28, 29]. However, if DRG codes were not sufficiently reflecting the differences in characteristics of patients, providers and health services, it might lead to too low payments for high complex cases, and too high for less-complex cases. Furthermore, DRG-coding is administratively complicated, mis-coding may occur in practice [30]. In Sweden, the SNBHW is responsible for linking DRG codes to the relevant costs. In general, a bottom-up approach was applied, incorporating all direct and indirect medical costs [31], including costs for all health professionals e.g., doctors’ and/or nurses’ fees, infrastructure, important medical equipment and installations, communication systems, or informatics.

The mean cost is an important summary statistic from both budgetary and social perspectives [29], however, cost data are also well known for their skewed distribution, therefore, it becomes challenging to choose an appropriate statistical method. Summary statistics such as median cost and non-parametric tests such as the Mann–Whitney* U test* were typically applied for non-normally distributed data. However, these analyses could only analyse whether the distribution differs between the groups and thus they are not recommended for economic evaluation [27, 29]. The Good Research Practices for Cost-Effectiveness Analysis Alongside Clinical Trials by the ISPOR RCT-CEA Task Force Report recommended that the arithmetic mean cost differences should be considered as the most appropriate and robust measure [27]. In most cases, bootstrap is an appropriate method to compare means and calculate confidence intervals [27]. We applied GLS based on the Gaussian and gamma distribution and adjusted for different factors, which gave more robustness in the results.

Various algorithms have been developed for mapping from EORTC QLQ-C30 to EQ-5D index, two studies have compared the different mapping algorithms and their prediction ability through external datasets [32, 33]. However, the conclusions were contradictory. While Frank et al. claimed that the choice of mapping algorithm might only have a small impact on the predicted utility and cost-effectiveness [32], Crott et al. found that the mapping algorithm might lead to underestimating both the mean and variance of the mapped EQ-5D utilities and the relationship between EORTC-QLQ-C30 scores and EQ-5D values were not stable across the different data sets. They suggested that mapping from EORTC-QLQ-C30 profiles to EQ-5D utilities using published algorithms should be performed with reservations [33]. In our study, the algorithm developed by Versteegh et al. [15] was used. The focus of our study was to compare the two arms in the clinical trial and, therefore, the choice of mapping algorithm would have a limited impact on the results. However, for future studies, we would recommend adding a generic utility measure, to avoid the potential problems caused by applying the mapping algorithm. The combination of both the generic instrument (estimating utility, enabling comparisons across disease) and condition-specific instruments (capturing health problems relevant for specific patients) would provide a better understanding of the patients’ QoL.

### Limitation

There is no nationwide register data for primary care and, therefore, the cost for primary care could not be included. This might lead to underestimation of health care costs in both arms. None of the existing mapping algorithms supports mapping to EQ-5D utility score based on Swedish preference. As health preference differs between countries, it is generally required to use country-specific value sets to calculate health utility [34]. For future studies in Sweden applying the EQ-5D instrument, we recommend using the Swedish value sets [35, 36].

## Conclusion

No significant differences were found in ICER between the two arms after 6 years of follow-up. The current study did not support that the ultra-hypo fraction treatment was more cost-effective than the conventional fraction treatment up to the sixth year of the trial.

## Supplementary Information

Below is the link to the electronic supplementary material.

Supplementary file1 (PDF 457 KB)

## Data Availability

Data sharing is not possible according to Swedish law.

## Code availability

All analyses were conducted using R.4.0.2.
