# Supplementary material for: Is ultra-hypo-fractionated radiotherapy more cost-effective relative to conventional fractionation in treatment of prostate cancer? A cost–utility analysis alongside a randomized HYPO-RT-PC trial
Source: Eur J Health Econ. 2022 May 19;24(2):237–46. doi: 10.1007/s10198-022-01467-5 (PMC9985558; doi:10.1007/s10198-022-01467-5)
Supplement: Supplementary file 1 — Supplementary file1 (PDF 457 KB) [file 10198_2022_1467_MOESM1_ESM.pdf]

Supplementary material

Is ultra-hypofractionated radiotherapy more cost-effective relative to conventional fractionation in treatment of prostate cancer? A cost-utility analysis alongside a randomized HYPO-RT-PC trial.

Figure 1. Costs (SEK) by ICD10 categorization at baseline and follow-ups, by treatment arms, UHF vs CF (for details of ICD 10, see S1)

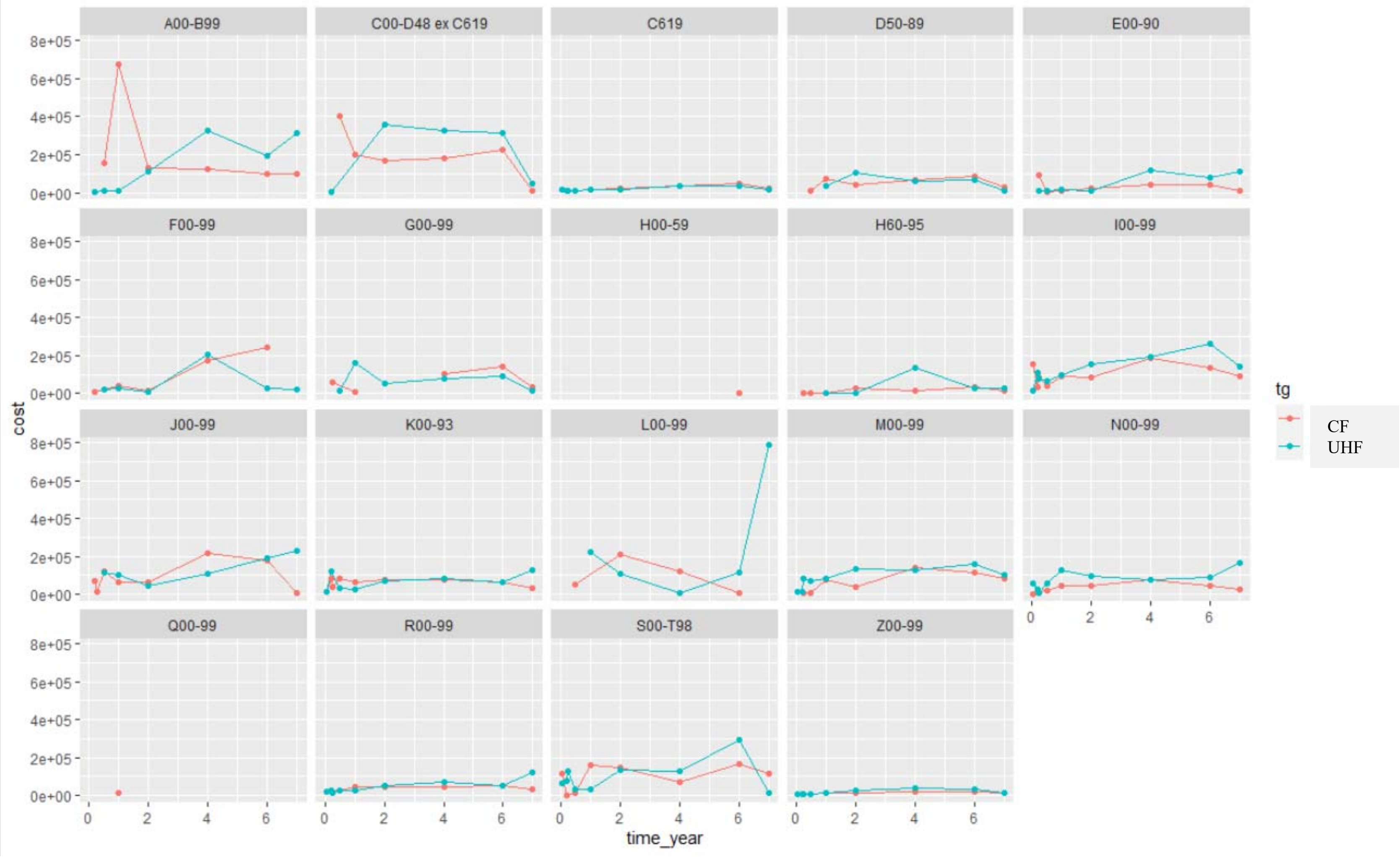

Figure 2-Distribution of cost data

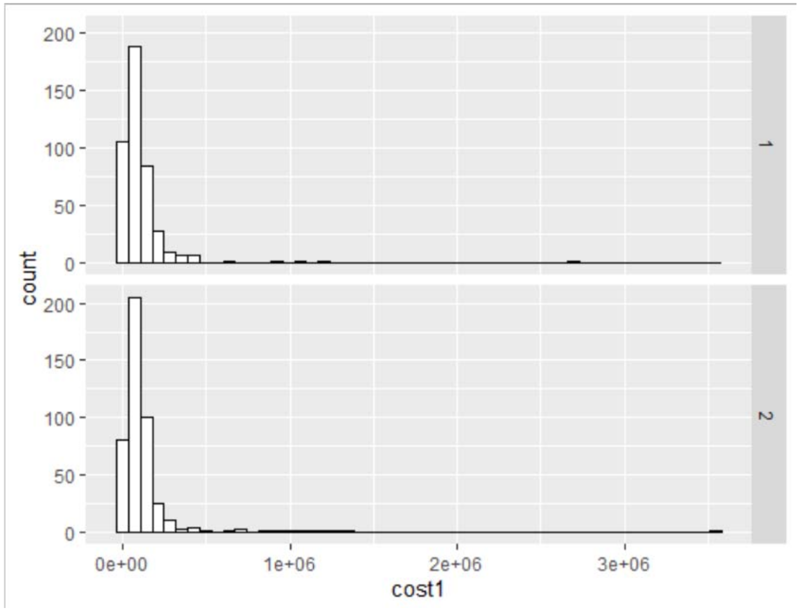

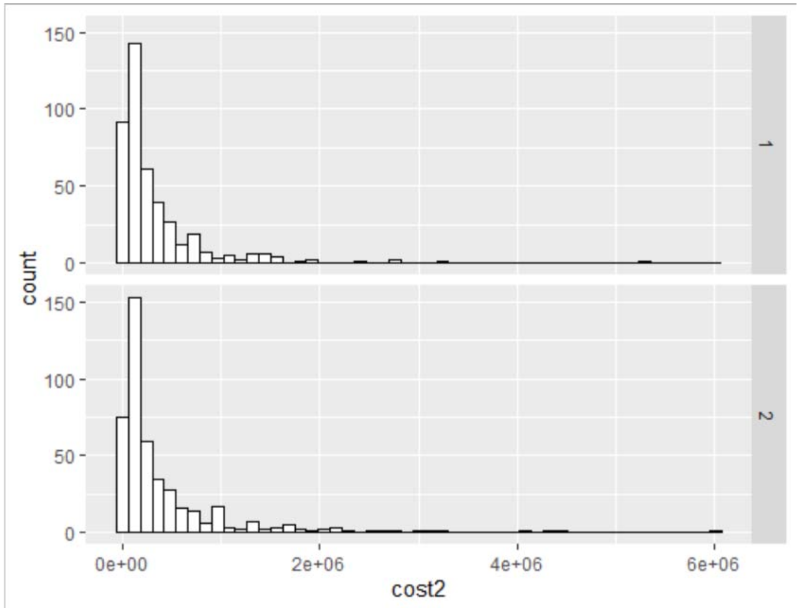

**Cost 1, including costs for prostate cancer and related disease (C619, N00-99 and Z00-99)**	**Cost 2, including costs for all the ICD categories**

Figure 3. Confidence interals for the incremental cost-effectiveness ratio (ICER) observed from 1000 bootstrap , for cost1 (including costs for prostate cancer and related disease only, C619, N00-99 and Z00-99) and cost 2 including costs for all ICD categories respectively

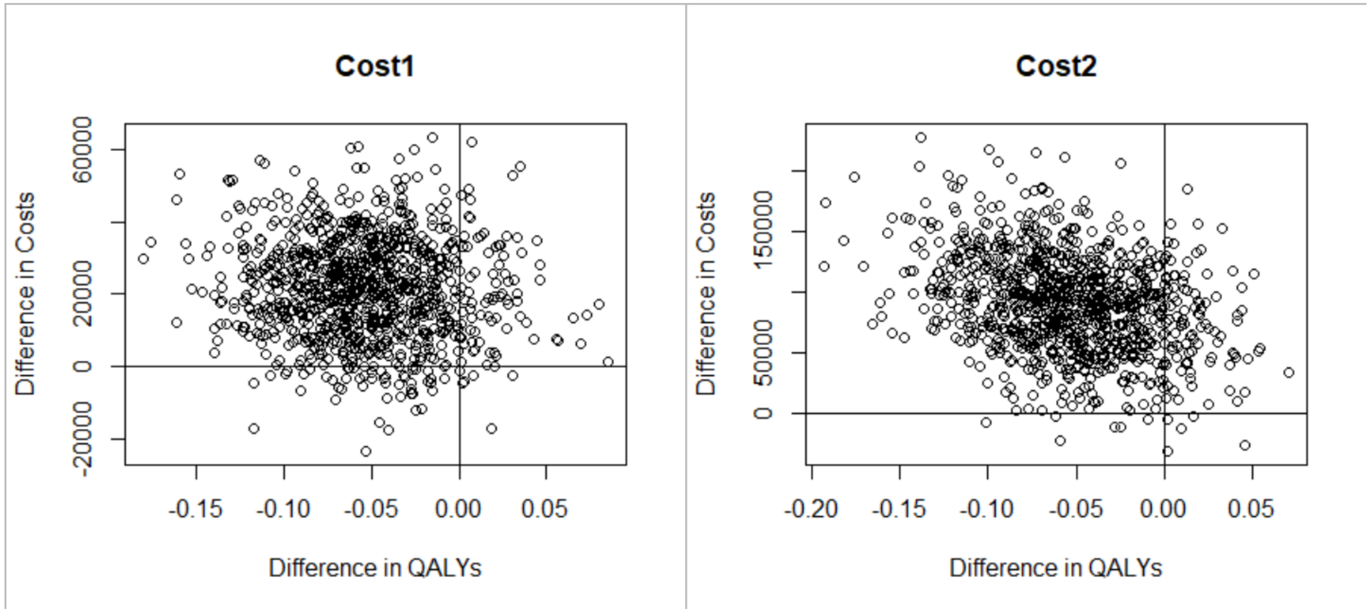

S1. Mean costs (SEK) by ICD category, by treatment arm

ICD category		CHF	UHF	Difference	p-Value[a]
A00-B99	Certain infectious and parasitic diseases	141806.3	139011.8	-2794.5	0.293
C00-D48 exclude C619	Tumors except prostate cancer	65207.1	97696.3	32489.1	0.185
C619	Prostate cancer	12392.5	11780.2	-612.3	0.505
D50-89	Diseases of the blood and blood-forming organs and certain disorders of the immune system	49450.8	52494.6	3043.8	0.873
E00-90	Endocrine disorders, nutritional disorders and metabolic disorders	22408.0	30182.3	7774.3	0.228
F00-99	Mental illness and syndrome and behavioral disorders	55694.3	45927.3	-9767.0	0.586
G00-99	Diseases of the nervous system	46527.7	67096.6	20568.9	0.117
H60-95	Diseases of the ear and mastoid committee	12219.2	55970.0	43750.8	0.408
**I00-99**	**Diseases of the circulatory system**	**65665.6**	**73695.0**	**8029.4**	**0.037**
J00-99	Respiratory diseases	73970.4	120432.1	46461.7	0.482
K00-93	Diseases of the digestive organs	48450.6	34912.5	-13538.1	0.857
L00-99	Skin and subcutaneous tissue disorders	85306.2	74511.8	-10794.4	0.358
M00-99	Diseases of the musculoskeletal system and connective tissue	29600.3	66368.8	36768.5	0.554
N00-99	Diseases of the urinary and genital organs	31069.2	41598.1	10528.9	0.125
R00-99	Symptoms, signs and abnormal clinical and laboratory findings not elsewhere classified	29509.4	40499.0	10989.6	0.289
S00-T98	Injuries, poisonings and certain other consequences of external causes	75015.2	82908.8	7893.6	0.321
**Z00-99**	**Factors of importance for the state of health and for contacts with the health service**	**9204.9**	**10976.6**	**1771.7**	**0.043**
Q00-99	Congenital malformations, deformities and chromosomal abnormalities	12801.0	0.0	12801.0	n.a.
H00-59	Diseases of the eye and nearby organs	4199.0	0.0	-4199.0	n.a.

Rows with shadows used for calculating costs for prostate cancer and related disease (C1)

S2, Costs occurred 2 years prior to the trial, by treatment arm

	Cost 3 (costs occurred 2 years prior to the trial)			
	CF (n=434)		UHF (n=445)	
	n	%	n	%
0 SEK	337	78%	324	73%
1-11962 SEK	71	16%	95	21%
11963-63030 SEK	26	6%	25	6%
